# Dimorphism of *Trichosporon cutaneum* and impact on its lipid production

**DOI:** 10.1186/s13068-019-1543-3

**Published:** 2019-08-29

**Authors:** Ya Wang, Riming Yan, Lijuan Tang, Libin Zhu, Du Zhu, Fengwu Bai

**Affiliations:** 1 State Key Laboratory of Microbial Metabolism & School of Life Science and Biotechnology, Shanghai Jiao Tong University, 800 Dongchuan Rd., Shanghai, 200240 China; 2 School of Life Science, Jiangxi Science and Technology Normal University, 605 Fenglin Rd., Nanchang, 330013 China; 3 Key Laboratory of Protection and Utilization of Subtropic Plant Resources of Jiangxi Province, School of Life Science, Jiangxi Normal University, 99 Ziyang Rd., Nanchang, 330022 China

**Keywords:** *Trichosporon cutaneum*, Dimorphic transition, Assimilable nitrogen, Lipid biosynthesis, Signal transduction

## Abstract

**Background:**

Compared to the oleaginous yeast *Yarrowia lipolytica*, *Trichosporon cutaneum* can metabolize pentose sugars more efficiently, and in the meantime is more tolerant to inhibitors, which is suitable for lipid production from lignocellulosic biomass. However, this species experiences dimorphic transition between yeast-form cells and hyphae during submerged fermentation, which consequently affects the rheology and mass transfer performance of the fermentation broth and its lipid production.

**Results:**

The strain *T. cutaneum* B3 was cultured with medium composed of yeast extract, glucose and basic minerals. The experimental results indicated that yeast-form morphology was developed when yeast extract was supplemented at 1 g/L, but hyphae were observed when yeast extract supplementation was increased to 3 g/L and 5 g/L, respectively. We speculated that difference in nitrogen supply to the medium might be a major reason for the dimorphic transition, which was confirmed by the culture with media supplemented with yeast extract at 1 g/L and urea at 0.5 g/L and 1.0 g/L to maintain total nitrogen at same levels as that detected in the media with yeast extract supplemented at 3 g/L and 5 g/L. The morphological change of *T. cutaneum* B3 affected not only the content of intracellular lipids but also their composition, due to its impact on the rheology and oxygen mass transfer performance of the fermentation broth, and more lipids with less polyunsaturated fatty acids such as linoleic acid (C18:2) were produced by the yeast-form cells. When *T. cutaneum* B3 was cultured at an aeration rate of 1.5 vvm for 72 h with the medium composed of 60 g/L glucose, 3 g/L yeast extract and basic minerals, 27.1 g (dry cell weight)/L biomass was accumulated with the lipid content of 46.2%, and lipid productivity and yield were calculated to be 0.174 g/L/h and 0.21 g/g, respectively. Comparative transcriptomics analysis identified differently expressed genes for sugar metabolism and lipid synthesis as well as signal transduction for the dimorphic transition of *T. cutaneum* B3.

**Conclusions:**

Assimilable nitrogen was validated as one of the major reasons for the dimorphic transition between yeast-form morphology and hyphae with *T. cutaneum*, and the yeast-form morphology was more suitable for lipid production at high content with less polyunsaturated fatty acids as feedstock for biodiesel production.

**Electronic supplementary material:**

The online version of this article (10.1186/s13068-019-1543-3) contains supplementary material, which is available to authorized users.

## Background

Biofuels produced from renewable biomass resources have been intensively studied to address concerns on the sustainable supply of petroleum-based fuels as well as the impact from the over-consumption of fossil fuels on environments, particularly greenhouse gas emissions and consequent climate changes [1, 2]. As one of the most important liquid biofuels, biodiesel has garnered great interest due to its advantages over conventional diesel fuel [3]. However, as the mono-alkyl esters of long-chain fatty acids, preferably methyl esters, biodiesel has been produced predominately so far from vegetable oils, which apparently cannot support its production at large scale in the future to alleviate dependence on petroleum-based diesel fuel, since vegetable oils are sources of edible oils for human being [4]. Microbial oils produced by oleaginous microbes from biomass, especially from lignocellulosic biomass, are non-edible oils, which potentially are sustainable feedstock for biodiesel production [5, 6]. Among various microorganisms, oleaginous yeasts seem more promising due to their fast growth and efficient accumulation of intracellular lipids at high content [7].

Nitrogen limitation created by the high ratio of C/N is one of the most common and effective strategies for lipid production by oleaginous yeasts such as *Yarrowia lipolytica* [8]: when nitrogen is deficient, the activity of adenosine monophosphate (AMP) deaminase is up-regulated for breaking down AMP to release ammonium for nitrogen-starved cells, which consequently disrupts the tricarboxylic acid (TCA) cycle at the point of isocitrate due to the low level of intracellular AMP, resulting in citrate accumulation for being excluded into cytosol by citrate-malate translocase; the citric acid can be further cleaved into acetyl-CoA and oxaloacetate by the ATP-citrate lyase, and the oxaloacetate is then converted into malate by malate dehydrogenase; the malate can be translocated into mitochondria by citrate-malate translocase or converted by malic enzyme into pyruvate through the cytosolic transhydrogenase cycle associated with the production of NADPH and CO_2_; the net acetyl-CoA and NADPH produced under the nitrogen limitation condition finally flow into the pathways for synthesizing triacylglycerols and fatty acids.

Yeasts may present different morphologies from unicellular or yeast-like cells to multicellular pseudohyphae or hyphae, and environmental conditions, particularly nutritional depletion, have a significant impact on their morphological switch. For pathogenic *Candida albicans*, the dimorphic transition between yeast-like cells and hyphae is a strategy evolved for survival during its commensal lifestyle with hosts [9], but for non-pathogenic yeasts, such a morphological shift would affect their product formation, due to its significant impact on the rheological properties, mixing and mass transfer performance of the fermentation broth under submerged culture conditions, particularly on dissolved oxygen (DO) that is critical for obligate aerobic species such as *Y. lipolytica* [10].

Morphological shift has been intensively studied for the brewing yeast *Saccharomyces cerevisiae* for more understanding on fundamentals in life science [11–13]. However, it is not significant from the viewpoint of process engineering and industrial applications, since it is less likely for this species to develop with hyphae under submerged culture conditions, although pseudohyphae may be observed. Environmental stimuli can induce the morphological shift of *Y. lipolytica* [14], and molecular mechanisms underlying this phenomenon have been explored to some extent [15], but there are still many unknowns to be elucidated for controlling its morphology properly to improve lipid production.

As a model of oleaginous yeasts, *Y. lipolytica* has been studied intensively for lipid production and also as a host for metabolic engineering to further improve its lipid production capacity [16, 17], but its wild-type strains cannot metabolize pentose sugars efficiently for lipid production and are less tolerant to inhibitors released during the pretreatment of lignocellulosic biomass [18]. *Trichosporon cutaneum* is another oleaginous yeast characterized by its broad substrate spectrum and tolerance to inhibitors [19–21], which is more suitable for lipid production from lignocellulosic biomass. We studied lipid production from cassava by the strain *T. cutaneum* B3 [22], and found it grew as a mixture of yeast-form cells and hyphae in the medium composed of yeast extract, peptone and glucose, during which nitrogen concentration might play an important role in inducing its dimorphic transition [23]. In this work, we further investigated the morphological transition of *T. cutaneum* B3 and its impact on lipid production as well as molecular mechanism underlying this phenomenon.

## Results and discussion

### Dimorphism of *T. cutaneum* B3 and its impact on lipid production

**Fig. 1:**
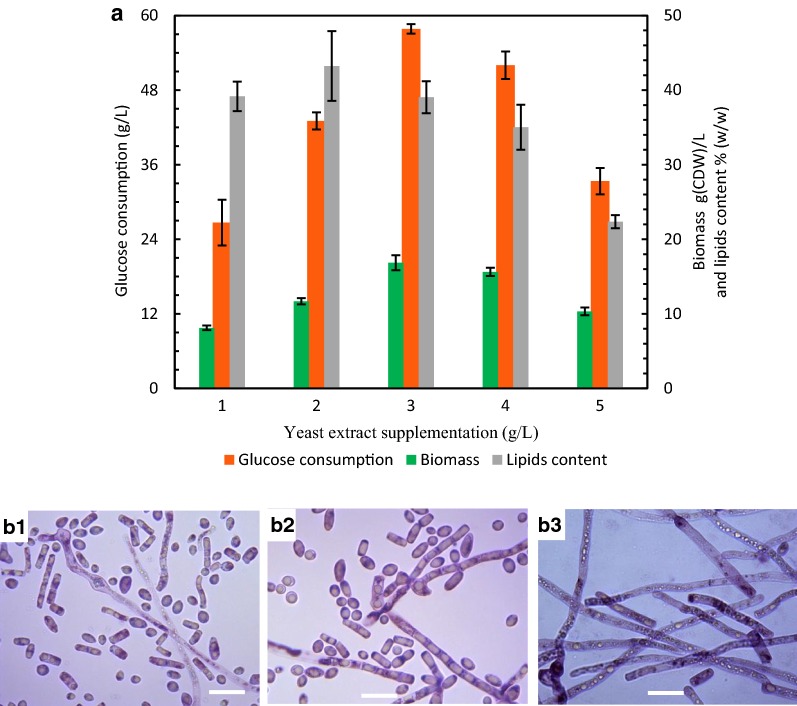
Results for the batch culture of *T. cutaneum* B3 in flasks with medium containing glucose of 60 g/L and yeast extract supplemented with different levels (**a**) and observation of yeast morphologies with a magnification of ×1000 for cultures with yeast extract supplemented at 1 g/L (**b1**), 3 g/L (**b2**) and 5 g/L (**b3)**, respectively. The bars represent 20 μm

Yeast growth and lipid synthesis are regulated by nutritional conditions, particularly nitrogen limitation, through which amino acid metabolism is regulated to redirect carbon flux to lipid synthesis [24]. Figure 1a shows the impact of yeast extract supplementation on glucose consumption, cell growth and lipid accumulation of *T. cutaneum* B3 under flask culture conditions. The highest lipid content of 43.2% based on dry cell weight (DCW) was obtained when 2 g/L yeast extract was supplemented, but only 43.0 g/L glucose was consumed with 11.7 g (DCW)/L biomass accumulated, and lipid production was calculated to be 5.0 g/L. As yeast extract supplementation was increased to 3 g/L, 57.8 g/L glucose was consumed, and 16.8 g(DCW)/L biomass was accumulated. Although lipid content was compromised to 39.1%, lipid production was increased to 6.6 g/L.

Yeast extract is rich with assimilable nitrogen. When more yeast extract is supplemented, yeast should grow better for more biomass to be accumulated and consequently more glucose consumed. However, when yeast extract was supplemented at 4 g/L and 5 g/L, respectively, *T. cutaneum* B3 grew poorly with less biomass accumulated, and glucose consumption was also compromised. To explore the reasons for this unusual phenomenon, yeast cells were sampled and observed under a microscope. As can be seen in Fig. 1b, *T. cutaneum* B3 grew with the morphology of hyphae when yeast extract was supplemented at 5 g/L instead of yeast-form cells observed when yeast extract was supplemented at 1 g/L, and a mixture of yeast-form cells and hyphae was observed when yeast extract was supplemented at 3 g/L. Hyphae development made the fermentation broth very viscous, which consequently deteriorated its mixing and oxygen mass transfer performance. As a result, the dimorphic transition of *T. cutaneum* B3 triggered by the supply of yeast extract at high concentration significantly affected its growth, glucose consumption and lipid production.

**Fig. 2:**
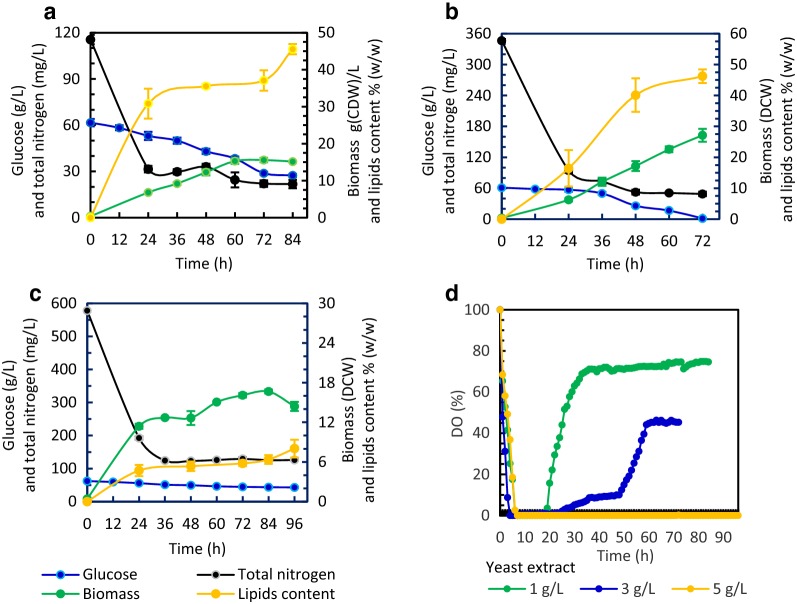
Consumption of glucose and total nitrogen and accumulation of biomass and lipids during the batch culture of *T. cutaneum* B3 within the fermenter using media supplemented with 60 g/L glucose and yeast extract at 1.0 g/L (**a**), 3.0 g/L (**b**) and 5.0 g/L (**c**), respectively, and DO profiles of the fermentation broth (**d**). The fermenter was operated at 28 ± 1 °C, pH 5.8 ± 0.5 and 200 rpm. An aeration of 3.0 L/min, equivalent to 1.5 vvm, was applied to the process

**Fig. 3:**
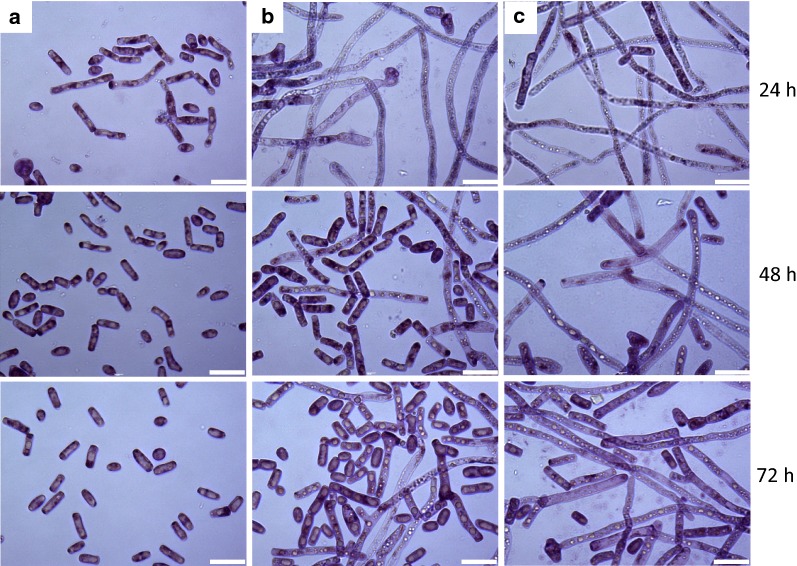
Morphological observation of *T. cutaneum* B3 under a magnification of ×1000 for culture with media composed of 60 g/L glucose and yeast extract supplemented at 1 g/L (**a**), 3 g/L (**b**) and 5 g/L (**c**). The bars represent 20 μm

For more understanding on the dimorphic transition observed under flask culture conditions and its impact on lipid production, *T. cutaneum* B3 was cultured within the bioreactor under different yeast extract supplementation conditions, and time courses were recorded for the consumption of glucose and total nitrogen, biomass accumulation, lipid production and the DO profile of the fermentation broth (Fig. 2). In addition, morphologies of *T. cutaneum* B3 developed at the early, middle and late stages of the culture were also observed (Fig. 3).

When yeast extract was supplemented at 1 g/L, total nitrogen was measured to be 115.5 mg/L, which was consumed quickly within 24 h with only 31.5 mg/L total nitrogen detected. The nitrogen starvation compromised glucose consumption and yeast growth, and 27.4 g/L glucose remained with 15.2 g(DCW)/L biomass accumulated, even when the fermentation time was extended to 84 h. Meanwhile, total lipids detected in the biomass were 45.5%. *T. cutaneum* B3 was grown predominately in the yeast-form morphology during the whole process, and all yeast cells were unicellular till the end of the culture (Fig. 3a). As yeast extract supplementation was increased to 3 g/L with total nitrogen increased to 346.5 mg/L, all glucose was consumed at 72 h, and biomass was accumulated to 27.1 g(DCW)/L with the lipid content increased to 46.2%, but hyphae were observed at the early stage when nitrogen was rich, which were transformed into yeast-form morphology gradually with the assimilation of the total nitrogen (Fig. 3b). When yeast extract supplementation was further increased to 5 g/L, both glucose consumption and yeast growth were significantly affected, since as high as 43.0 g/L glucose was left with only 14.4 g(DCW)/L biomass accumulated, and in the meantime intracellular lipid content was drastically decreased to 8.0%, even when the fermentation time was extended to 96 h. The reason for this phenomenon was the development of hyphae under the nitrogen-rich condition (Fig. 3c), making the fermentation broth extremely viscous and its oxygen mass transfer performance was deteriorated, which was confirmed by the DO profile highlighted in Fig. 2d.

For microbial lipid production under aerobic conditions, high lipid productivity and yield are the most important factors from the viewpoint of bioprocess engineering to save energy input for aeration and feedstock consumption for lipid biosynthesis. When 3 g/L yeast extract was supplemented, lipid productivity was improved to 0.174 g/L/h, and lipid yield was increased to 0.21 g/g since more biomass with high lipid content was accumulated. These results are better than those achieved previously in other oleaginous yeasts: ~ 0.1 g/L/h and 0.11 g/g for *Y. lipolytica* [16] and 0.06 g/L/h and 0.17 g/g for *Lipomyces starkeyi* [25]. When genetic modifications were applied with *Y. lipolytica* to decouple the onset of lipid production from nitrogen limitation, its lipid productivity would be enhanced substantially [26]. Similar strategies should be applicable for *T. cutaneum* to further improve its lipid productivity.

Our experimental results demonstrated that *T. cutaneum* B3 presented two significantly different morphologies under submerged fermentation conditions: yeast-form or unicellular cells and hyphae, and nitrogen levels might induce the dimorphic transition. To validate such a speculation, we designed media supplemented with 1 g/L yeast extract to provide micronutrients, but nitrogen was supplied mainly by urea, which was supplemented at 0.5 g/L and 1.0 g/L, respectively, making the total nitrogen equivalent to that detected in the media supplemented with yeast extract at 3 g/L and 5 g/L. The experimental results supported our speculation for the impact of nitrogen supply on the dimorphic transition of *T. cutaneum* B3 during submerged fermentation (Additional file 1: Fig. S1). In addition to nitrogen limitation that has been intensively studied for lipid biosynthesis with different oleaginous microorganisms, oxygen mass transfer is also very important for yeast growth and lipid biosynthesis, which can be affected significantly when oleaginous yeasts grow with different morphologies. No doubt, yeast-form morphology characterized by unicellular cells is preferred for better oxygen mass transfer due to its less impact on the rheological properties of the fermentation broth, particularly when oxygen demand is vigorous in the middle stage with robust metabolism and late stage with high cell density.

### Composition of fatty acids in lipids produced by *T. cutaneum* B3

Composition of fatty acids in lipids affects biodiesel properties such as oxidation stability and cold filter plugging point [27]. As shown in Table 1, long-chain fatty acids were synthesized by the yeast-form cells and hyphae of *T. cutaneum* B3, with palmitic acid (C16:0), stearic acid (C18:0), oleic acid (C18:1) and linoleic acid (C18:2) as major components. Although the ratio of C16 and C18 fatty acids in the lipids was 93–95%, the percentages of oleic acid and linoleic acid were different when *T. cutaneum* B3 grew with different morphologies. While oleic acid and linoleic acid in the lipids produced by the yeast-form cells were about 55% and 5%, respectively, much less oleic acid (~ 35%) and more linoleic acid (~ 20%) were produced by the hyphae, making the percentages of monounsaturated fatty acids (MUFAs) and polyunsaturated fatty acids (PUFAs) in lipids produced by *T. cutaneum* B3 substantially different. Although more PUFAs are beneficial for the low cold filter plugging point, they compromise other fuel properties of biodiesel such as cetane number and oxidation stability [28]. Therefore, the culture of *T. cutaneum* B3 should be controlled predominately with the yeast-form morphology.

**Table 1:** Composition of fatty acids in lipids produced by *T. cutaneum* B3 grown in the bioreactor when the medium was supplemented with yeast extract at 1, 3 and 5 g/L, respectively

Yeast extract supplementation (g/L)	Ratio of fatty acids (%, w/w)										
	C14:0	C16:0	C16:1	C17:0	C18:0	C18:1	C18:2	C18:3	SFA	MUFA	PUFA
1	0.197	19.765	0.177	0.064	15.120	56.859	4.867	0.070	35.146	57.036	4.937
3	0.266	20.368	0.219	0.059	11.049	57.089	6.709	0.113	31.742	57.308	6.822
5	0.787	20.668	0.522	0.087	15.661	36.077	19.278	0.646	37.203	36.599	19.924

SFA, saturated fatty acids; MUFA, monounsaturated fatty acids; PUFA, polyunsaturated fatty acids

### Comparative transcriptome analysis for genes related to lipids biosynthesis

Since more lipids were accumulated when *T. cutaneum* B3 was grown in yeast-form morphology, comparative transcriptome analysis for genes related to lipid biosynthesis was performed for yeast cells cultured in the media supplemented with 1 g/L and 5 g/L yeast extract, which were sampled during their vigorous growth at 24 h.

A total of 3784 differentially expressed genes (DEGs) were screened with the thresholds: FDR < 0.05 and − 1 ≥ Log_2_R ≥ 1, in which 2165 genes were up-regulated, and 1619 were down-regulated (Additional file 1: Fig. S2). To validate the reliability of the transcriptome analysis, 10 genes related to lipid biosynthesis, signal transduction for the dimorphic transition and other pathways to be analyzed in the future were selected for the qPCR analysis (Additional file 1: Fig. S3), and the results were in accordance with their comparative transcriptome analysis. Gene ontology (GO) was applied for the DEGs, which categorized them mainly into biological process, cellular component and molecular function (Additional file 1: Fig. S4). To further elucidate the biological functions of the DEGs, KEGG pathway enrichment was performed, through which more than 200 pathways were enriched, and pathways related mainly to lipid metabolism and signal transduction for stress response are highlighted (Additional file 1: Fig. S5). As can be seen, most up-regulated gene in the yeast-form cells were related to ribosome biogenesis in eukaryotes, carbon metabolism, amino acid metabolism such as alanine, aspartate and glutamate and fatty acid metabolism. Ribosome is the factory where proteins are synthesized based on the information of mRNA. Among 120 genes annotated to the ribosome pathway, 55 genes were annotated for encoding ribosomal proteins, which were up-regulated at least twofold in the yeast-form cells, indicating a vigorous process for biosynthesis to facilitate fatty acid metabolism.

### Central carbon metabolism for lipid biosynthesis in *T. cutaneum* B3

**Fig. 4:**
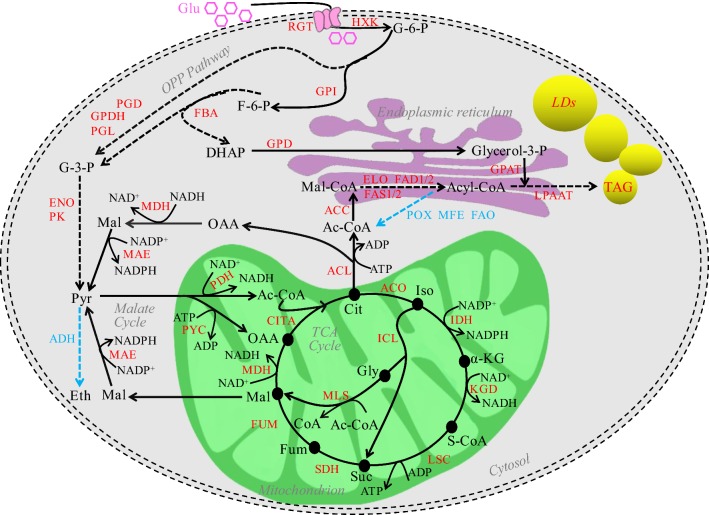
Central carbon metabolism of *T. cutaneum* B3 for lipid synthesis. Up- and down-regulation of genes encoding key enzymes and transcriptional regulators are highlighted in red and blue colors, respectively. Dashed arrows indicate multiple enzymatic steps in the metabolic pathway. ACC, acetyl-CoA carboxylase; Ac-CoA, acetyl-CoA; ACL, ATP-dependent citrate synthase; ACO, aconitase; ADH, acetaldehyde dehydrogenase; α-KG, α-ketoglutaric acid; Cit, citrate; CITA, citrate synthase; DHAP, dihydroxyacetone phosphate; ELO, fatty acid elongase; ENO, enolase; Eth, ethanol; F-6-P, frucose-6-phosphate; FAD1, delta-9 fatty acid desaturase; FAD2, delta-12 fatty acid desaturase; FAO, long-chain-alcohol oxidase; FAS1/FAS2, fatty-acid synthase complex protein 1/2; FBA, fructose-bisphosphate aldolase; Fum, fumaric acid; FUM, fumarate hydratase; Glu, glucose; Gly, glyoxylate; G-3-P, glyceraldehyde-3-phosphate; G-6-P, glucose-6-phosphate; Glycerol-3-P, glycerol-3-phosphate; GPAT, glycerol-3-phosphate acyltransferase; GPD, Glycerol-3-phosphate dehydrogenase; GPDH, glucose-6-phosphate dehydrogenase; GPI, glucose-6-phosphate isomerase; HXK, hexokinase; IDH, isocitrate dehydrogenase; ICL, isocitrate lyase; Iso, isocitric acid; KGD, α-ketoglutarate dehydrogenase; LDs, lipids droplet; LPAAT, lyso-phosphatidic acid acyltransferase; LSC, succinyl-CoA synthetase; MAE, malic enzyme; Mal, malate; Mal-CoA, malonyl-CoA; MDH, malate dehydrogenas; MFE, β-oxidation multifunctional enzyme; MLS, malate synthase; OAA, oxaloacetate; OPP, Pathway oxidative pentose phosphate pathway; PDH, pyruvate dehydrogenase; PGD, 6-phosphogluconate dehydrogenase; PGL, 6-phosphogluconolactonase; PK, pyruvate kinase; POX, acyl-CoA oxidase; PYC, pyruvate carboxylase; PYR, pyruvate; RGT, high-affinity glucose transporter; SDH, succinate dehydrogen; S-CoA, succinyl-CoA; Suc, succinic acid; TAG, triacylglycerol; TCA, tricarboxylic acid cycle

Based on the comparative transcriptome analysis, central carbon metabolism for lipid biosynthesis was developed for *T. cutaneum* B3 (Fig. 4). Glucose is an easily assimilable carbon source for most microorganisms. For *T. cutaneum* B3, *c25305_g1*, *c11289_g1* and *c29061_g1* encoding high-affinity glucose transporter RGT1/RGT2, were up-regulated with 2.14, 2.29 and 3.14 folds, respectively, making glucose transported from the bulk medium into cytosol more efficiently for being metabolized into pyruvate through the Embden–Meyerhof–Parnas (EMP) pathway, and the pyruvate was further converted into mitochondrial acetyl-CoA, which was transported into mitochondria to fuel the tricarboxylic acid cycle (TCA). Genes related to the EMP pathway such as *c11598_g1*, *c10066_g1*, *c11302_g2* and *c11526_g1* encoding hexokinase (HXK), fructose-bisphosphate aldolase (FBA), enolase (ENO) and pyruvate kinase (PK) were up-regulated, indicating an active glucose metabolism shunt with the yeast-form cells under the nitrogen-limit condition compared to the hyphae induced by the nitrogen-rich condition. However, the TCA pathway was interrupted at the point of isocitrate for citrate to be accumulated and excluded from mitochondria into cytosol where ATP-dependent citrate lyase (ACL) cleaved it into acetyl-CoA and oxaloacetate. While the acetyl-CoA was directed to lipid biosynthesis, the oxaloacetate was cleaved by malic dehydrogenase (MDH) into malate, which was further converted into pyruvate by malic enzyme (MAE) for additional pyruvate to be directed to the citrate-pyruvate cycle. This pathway is not common in typical oleaginous yeasts such as *Y. lipolytica*, but was reported in oleaginous fungi *Mucor circinelloides* and *Mortierella alpina* [29], which seems also work with *T. cutaneum* B3.

**Fig. 5:**
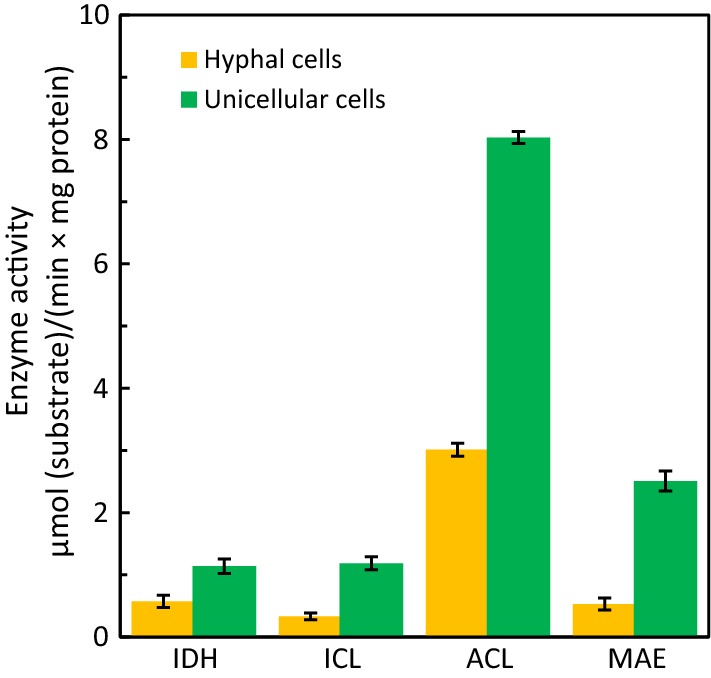
Activities of key enzymes for lipid biosynthesis in yeast-form cells and hyphae of *T. cutaneum* B3. IDH, isocitrate dehydrogenase; ICL, isocitrate lyase; ACL, ATP citrate lyase and MAE, malic enzyme

The activities of key enzymes including ACL, MAE, isocitrate dehydrogenase (IDH) and isocitrate lyase (ICL) were measured, and an increase of more than two times with their activities was observed in the yeast-form cells (Fig. 5), particularly for ACL and MAE, which is consistent with the comparative transcriptome analysis for genes encoding these enzymes as well as the central carbon metabolism highlighted in Fig. 4 for lipid biosynthesis in *T. cutaneum* B3. For example, the activity of ICL was improved properly to provide malate within mitochondria to complete the citrate-pyruvate cycle. It is worth noting that the enhanced activity of IDH observed with the yeast-form cells of *T. cutaneum* B3 seems not consistent with previous results, since it facilitates the downstream conversion of isocitrate, and thus is not favorable for citrate accumulation, but such an effect would be compromised by the shortage of AMP under nitrogen depletion conditions [8]. However, the high activity of IDH might indicate that *T. cutaneum* B3 presents a more robust TCA cycle compared to other oleaginous yeast species for energy production.

The transcription of many genes related to the biosynthesis of TAGs including *ACL*, *ACC*, *FAS1*, *FAS2*, *FAD1*, *FAD2* and *ELO* were remarkably up-regulated when *T. cutaneum* B3 was grown in yeast-form under the nitrogen limitation condition. On the other hand, all identified genes related to the degradation of fatty acids via β-oxidation were down-regulated for lipid accumulation.

### Signaling pathways involved in the dimorphic transition

Dimorphic transition between yeast-form cells and hyphae has been studied intensively with *C. albicans* to understand its biofilm formation through the branching of hyphae for virulence and drug resistance, particularly its hyphae development under nutrient limitation conditions for better colonization onto hosts [30, 31]. *Y. lipolytica* also experiences morphological changes during culture, which might be induced by various environmental factors such as physicochemical parameters including pH, temperature and DO, nutritional conditions and mechanical and hydrodynamic shearing within fermenters created by agitation and mixing [14]. However, we observed that *T. cutaneum* B3 switched to hyphal growth under nitrogen-rich conditions when yeast extract was supplemented at relatively high dosages, which seems not being reported previously for *Y. lipolytica* and other yeasts.

**Fig. 6:**
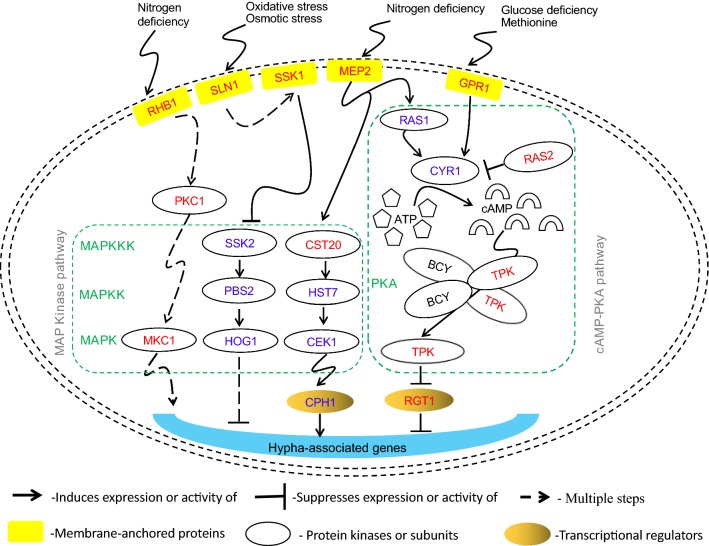
Signal transduction pathways and transcriptional regulators affecting the filamentous growth of dimorphic pathopoiesis fungus in response to environmental conditions, which were reconstructed based on the predicted morphological shift in *C. albicans* [9, 38, 40, 42]. The red and blue colors highlight the homologous genes significantly up- and down-regulated in *T. cutaneum* B3. Genes related to hyphae development might be activated by the transcription factor CPH1 thought the CEK1 MAPK pathway (MAPKK kinase CST20 and MAPK kinase HST7) or other two MAPK pathways through kinase MKC1 and HOG1. Oxidative and osmotic stresses are sensed by a two-component system with sensing proteins SLN1 and SSK1, which in turn suppress kinases SSK2 and PBS2 to trigger the high osmolarity glycerol (HOG) MAPK pathways. Nitrogen starvation is sensed by RHB1, a homologs of the small G protein RHEB with the RAS superfamily, which in turn activates the protein kinase PKC1 and MAPK kinase MKC1 in the MKC1 MAPK pathways. MEP2, a methylamine permease, may also sense nitrogen starvation to activate both the MAPK pathway and the cAMP-PKA pathway. Adenylyl cyclase (CYR1) not only responds to RAS1/RAS2 under nitrogen starvation condition, it is also activated in response to G-protein GPR1, which are activated by glucose deficiency and the presence of methionine. Protein kinase A (PKA) comprises of regulatory (BCY) and catalytic subunits (TPK), and TPK suppresses the transcription factor RGT1 and in turn suppresses the expression of hyphal-inducing genes

Environmental stimuli need to be sensed through signaling pathways, which have been investigated for hyphal development in *C. albicans* [32]. Mitogen-activated protein kinase (MAPK) pathways are key mediators for signaling in yeast under stressful conditions, which are characterized by a three-tiered module composed of MAPK kinase (MAPKKK), MAPK kinase (MAPKK) and MAPK itself [33]. Protein kinase A (PKA) is a family of enzymes, and the cyclic adenosine monophosphate (cAMP) dependent PKA (cAMP-PKA) pathway is another major transducer for signal sensing in *C. albicans* as a central regulator for morphological transition, in which cAMP can bind onto the regulatory subunit of PKA to release its catalytic subunit [34, 35]. Comparative transcriptome analysis indicates that *T. cutaneum* B3 may mediate its response to nitrogen starvation and dimorphic transition through both the MAPK cascade and the cAMP-PKA pathway, since many genes regulate these signaling processes for stress response were up-regulated, which are highlighted in Fig. 6. For example, *c11565_g1* and *c10952_g1* were over-expressed at 1.38- and 0.58-fold, respectively, under the nitrogen limitation condition. Sequence analysis indicates that *c11565_g1* and *c10952_g1* encode proteins that are homologous to CST20 and MEP2. In *C. albicans*, CST20 is one of the regulatory proteins for *CPH1*, and MEP2 is one of methylamine permeases to facilitate ammonium transport, enabling cell growth when ammonium is present at low concentration as only available nitrogen source, which also has a role in inducing filamentous growth through activating the transcription factor CPH1 and the CPH1-dependant MAPK cascade [36].

In addition, genes *c12106_g1* and *c16345_g1* were up-regulated at 0.58- and 3.36-folds, which are homologous to *RHB1* and *PKC1* in *C. albicans*, encoding RHB1, a homolog of the small G protein RHEB with the RAS superfamily in eukaryotic organisms to control various physiological processes including nitrogen starvation-induced morphogenesis and cell wall integrity [37], and the protein kinase PKC1. While RHB1 can activate both the MAPK cascade and the cAMP-PKA pathway in *C. albicans*, PCK1 regulates the MAPK signaling for stress response [38, 39]. Another differentially expressed genes are *c11060_g1* and *c18673_g1*, which were up-regulated at 1.07- and 1.58-folds, respectively, in the yeast-from cells. These genes are homologs to *SLN1* and *SSK1* in *C. albicans*, which encode the histidine kinase SLN1 and its regulating protein SSK1 to activate the HOG (high osmolarity glycerol) MAPK pathway through the *SLN1* branch for sensing not only osmotic pressure but also other signals, particularly a repression on filamentation for yeast cells to grow with unicellular morphology [40].

Small guanine nucleotide-binding proteins (G-proteins or GTPases) are highly conserved master regulators as a switch for signaling pathways to control fungal morphogenesis, which can be activated through binding with guanosine triphosphate (GTP) and deactivated when binding with guanosine diphosphate (GDP) released during GTP hydrolysis [41]. RAS GTPases including RAS1 and RAS2 encoded by *RAS1* and *RAS2* are conserved in *C*. *albicans* for regulating its multiple traits including yeast-hyphae transition through the cAMP-PKA pathway [42]. On the other hand, GPR1 encoded by *GPR1* is a G-protein-coupled receptor, which functions on the upstream of the cAMP-PKA pathway and affects its function in *C. albicans* [43]. The catalytic subunits of PKA in *S. cerevisiae* are encoded by *TPK1*, *TPK2* and *TPK3*, but there are two isoforms TPK1 and TPK2 encoded by *TPK1* and *TPK2* as the catalytic subunits of PKA in *C*. *albicans* [44, 45]. In *T. cutaneum* B3, *c9021_g1*, *c9875_g1* and *c10736_g1* are homologous to *GPR1*, *RAS2* and *TPK3*, which were overexpressed in the yeast-form cells at 1.14-, 1.58- and 1.63-folds to regulate the cAMP-PKA signaling pathway and consequently to repress the development of hyphae.

## Conclusions

In the study, we provided insights on the morphogenesis and lipogenesis of *T. cutaneum* B3. Nitrogen level was experimentally validated to be a key factor for triggering its dimorphic transition, and consequently affected its lipid production. Moreover, the morphological change also affected lipid composition, and the yeast-form cells synthesized more lipids with composition suitable for biodiesel production. Comparative transcriptome analysis performed under the nitrogen limitation and nitrogen-rich conditions identified genes differentially expressed for central carbon metabolism and lipid biosynthesis as well as signaling pathways for the dimorphic transition, which potentially can be used to guide strain development and process optimization for more efficient production of lipids by *T. cutaneum*.

## Materials and methods

### Strain, media and culture

*T. cutaneum* B3 was screened through mutation and deposited at China Center for Type Culture Collection (CCTCC) with the reference number of M2010076. The pure culture was transferred monthly on slant composed of (g/L): glucose 20, yeast extract 10, peptone 10 and agar 20, which was incubated at 30 °C for 48 h, and then collected and maintained at 4 °C as stock.

Medium for seed culture contained (g/L): glucose 40, yeast extract 3.0, KH_2_PO_4_ 0.75 and MgSO_4_ 0.4. Nitrogen-limited medium (NLM) contained (g/L): glucose 60, yeast extract 1.0, KH_2_PO_4_ 0.75 and MgSO_4_ 0.4. Nitrogen-moderate medium contained (g/L): glucose 60, yeast extract 3.0, KH_2_PO_4_ 0.75 and MgSO_4_ 0.4. Nitrogen-rich medium (NRM) contained (g/L): glucose 60, yeast extract 5.0, KH_2_PO_4_ 0.75 and MgSO_4_ 0.4. The pH of all media was 5.8 ± 0.05 after sterilization.

Flask culture: A loopful of inoculum was removed from the stock and inoculated into 250 mL flask containing 60 mL seed medium, which was incubated in an orbital shaker at 200 rpm and 28 °C for 2 d. The seed culture of 10 mL with ~ 5 × 10^6^ cells/mL was inoculated into 500 mL flask containing 120 mL fermentation medium, which was incubated at 200 rpm and 28 °C for lipid production.

Bioreactor culture: Batch culture was performed within the 5-L fermenter (Biotech-5BG-4, Baoxing Bioengineering Co., Inc., Shanghai, China) with a working volume of 2.0 L. Seed culture of 120 mL containing about 5 × 10^6^ cells/mL was inoculated into the fermenter. The culture was performed at 200 rpm, 28 ± 1 °C and pH 5.8 ± 0.5 controlled automatically by adding 8 M NaOH. A constant aeration of 3.0 L/min, equivalent to 1.5 vvm, was applied, and the DO in the fermentation broth was monitored.

### Observation of yeast morphologies

The morphologies of yeast cells sampled from the flask and bioreactor were observed under the digital light microscope (Motic BA310 digital, China) equipped with the built-in 3.0 Mega Pixel Digital Camera (Ted Pella, Inc., Redding, CA, USA) and Motic images plus 2.0 software. Samples were loaded onto slides, and dried at 60 °C for 3 min, which were stained for 2 min by methylene blue, and washed twice with deionized water for observation with the microscope.

### Analysis of biomass, glucose, total nitrogen and lipids

Yeast biomass was measured in DCW. Sample of 30 mL was centrifuged for 10 min at 10,000 rpm. The pellet was collected and washed twice with distilled water, which was dried at 80 °C until constant weight for balancing. The residual glucose was determined by the dinitrosalicylate method [46]. The total nitrogen content was determined following the National Food Safety Standard Determination of Protein in Foods (GB 5009.5–2010, China). Lipids were extracted from yeast cells using solvent composed of chloroform and methanol at 2:1 (v/v) [47]. Extracted lipids were transesterified into fatty acid methyl esters (FAME) according to the method of National Food Safety Standard Determination of Fatty Acids in Foods (GB5009.168–2010, China), which were quantified by gas chromatography (GC-2010Plus, Shimadzu) with a flame ionization detector (FID) operated at 260 °C and CD-2560 capillary column (100 m × 0.25 mm, ANPEL Inc., Shanghai, China) packed with dicyanopropyl polysiloxane. The column temperature was maintained at 130 °C for 15 min, and then increased from 130 to 240 °C at a rate of 4 °C/min for about 30 min.

### Determination of enzyme activities

Cell free extract was prepared as described by Bellou et al. [48]. Briefly, yeast cells were washed twice with 50 mM Na_2_HPO_4_/KH_2_PO_4_ buffer (pH 7.5), and re-suspended at a ratio of 1 mL buffer per 0.5 g wet biomass, which were disrupted on ice using sonicator (Scientz, Ningbo, China), and the debris was removed through centrifugation. The supernatant was collected and filtered through 0.2 μm membrane, and the activities of IDH (EC 1.1.1.42), ACL (EC 4.1.3.8), MAE (EC 1.1.1.40) and ICL (EC 4.1.3.1) were determined, respectively, according to methods developed previously [49–52]. The protein content was determined by the Coomassie blue staining method [53]. The analytical results were an average of triplicate measurements.

### RNA extraction, library construction and sequencing

Yeast-form cells and hyphae of *T. cutaneum* B3 were sampled for RNA extraction and quantitative real-time PCR (qRT-PCR) analysis. Total RNA was extracted from the sample by the TRIzol reagent kit from Invitrogen (Carlsbad, CA, USA), and the concentration and purity of the total RNA were assessed by OD_260_/OD_280_ using the spectrophotometer (Nanodrop 2000). mRNA was purified from the total RNA using poly-T oligo-attached magnetic beads, and sample with RNA integrity number values above 8 was used for library construction. Construction of the cDNA libraries and their sequencing were performed by Majorbio, Shanghai, China with Illumina Hiseq 4000, and 150 bp paired-end reads were generated for de novo assembly, and sequenced raw reads were deposited at the database of Sequence Read Archive (SRA) with the accession number of PRJNA480207. The raw data in FASTQ format were processed and transformed into clean and high-quality data by removing reads containing adapter, poly (N) tail and low-quality sequence. DEGs were screened by FDR ≤ 0.05 and − 1 ≥ Log_2_R ≥ 1 (R: the ratio of gene expression under the NLM and NRM conditions).

Gene annotation and functional analysis were performed based on the NCBI reference sequence (RefSeq) database (http://www.ncbi.nlm.nih.gov/RefSeq/), the Pfam protein families database (http://pfam.wustl.edu/), the clusters of orthologous groups of proteins (COG) database (http://www.ncbi.nlm.nih.gov.COG), the Swiss-Prot protein sequence database (https://web.expasy.org/groups/swissprot/), the Kyoto Encyclopedia of Genes and Genomes (KEGG) database (http://www.genome.jp/kegg/) and the Gene Ontology (GO) database (http://www.geneontology.org/). GO enrichment analysis of the DEGs was implemented using the software Goatools (https://github.com/tanghaibao/GOatools) and the agriGO database (http://bioinfo.cau.edu.cn/agriGO/index.php). Singular enrichment analysis was used to identify significantly enriched GO terms compared with the background genome. The KEGG database was used to perform analysis for the significantly enriched pathways with the DEGs compared to the whole genome background.

### qRT-PCR analysis

To validate the date from the RNA-seq analysis, qRT-PCR was performed for 10 selected genes using the SYBR Premix ExTaqTM (TaKaRa, Dalian, China) on the ABI 7500 real-time PCR system (Applied Biosystems, Foster City, CA, USA). Primers for the qRT-PCR analysis were designed using Primer Primer web version 6.0.0 (Premier Biosoft Inc., CA), and the information of those primers are listed in Additional file 1: Table S1. Glyceraldehyde-3-phosphate dehydrogenase (GAPDH) was used as the housekeeping gene based on its relatively constant expression. PCR conditions were: pre-denaturation at 95 °C for 10 min, denaturation at 95 °C for 10 s, followed by 40 cycles of amplification (95 °C for 15 s and 60 °C for 34 s). Triplicate was applied for biological and technical runs for all reference and selected genes. Gene expression was calculated using the 2^−ΔΔCt^ method [54].

## Additional file

**Additional file 1.** Additional figures and tables.

## Data Availability

The strain used in this work and raw data of the transcriptome analysis were deposited, which are publically available. All other data generated or analyzed are included in this article and its additional materials.
