# Supplementary material for: Dimorphism of Trichosporon cutaneum and impact on its lipid production
Source: Biotechnol Biofuels. 2019 Aug 29;12:203. doi: 10.1186/s13068-019-1543-3 (PMC6714079; doi:10.1186/s13068-019-1543-3)
Supplement: Supplementary file 1 — Additional file 1. Additional figures and tables. [file 13068_2019_1543_MOESM1_ESM.docx]

**Additional File 1**

B1

A1

C


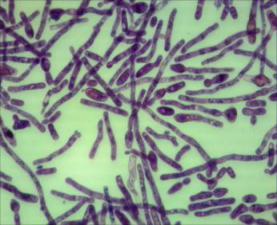

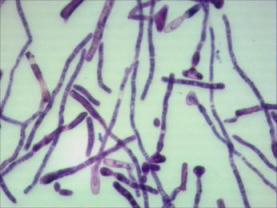

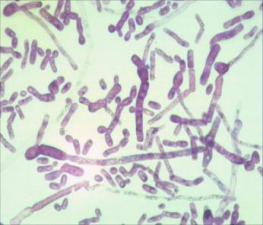

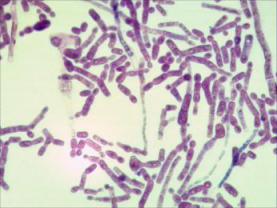

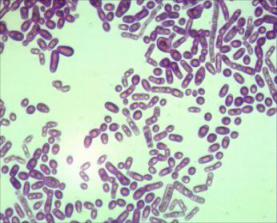

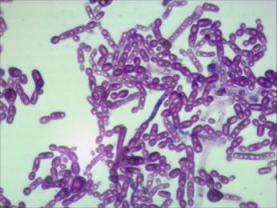

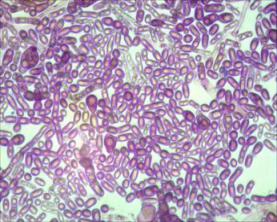

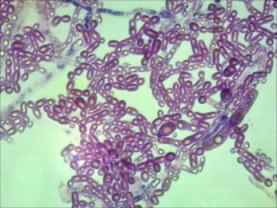

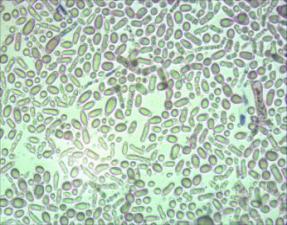

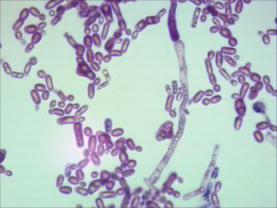


**A2**

**B2**

24 h

48 h

72 h

84 h

96 h

**Additional Fig. S1** Consumption of glucose (●) and total nitrogen (●), yeast growth (●), lipid accumulation (●) and morphologies of yeast cells observed under a magnification of X 1000 during the batch culture of *T. cutaneum* B3 within the bioreactor using media containing 60 g/L glucose, 1 g/L yeast extract and urea supplemented at 0.51 g/L (A1 and A2) and 1.0 g/L (B1 and B2) as well as DO profiles of the fermentation broth (C). The bars represent 20 μm.


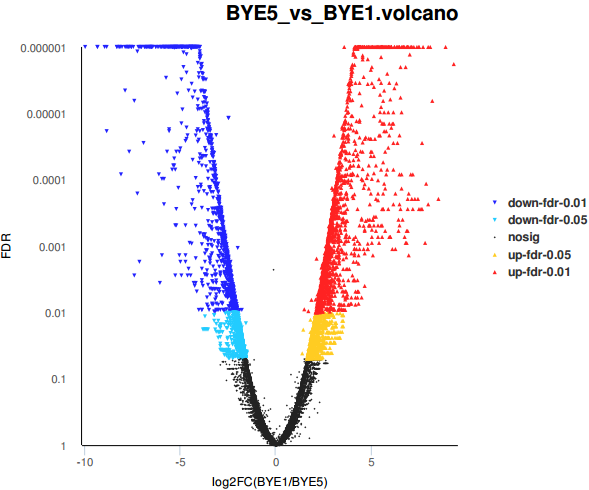


FDR

Log_2_FC

**Additional Fig. S2**. Overview for the transcriptome profiling of differentially expressed genes of *T. cutaneum* B3 cultured for 24 h using the medium supplemented with 1 g/L and 5 g/L yeast extract for unicellular morphology (BYE1) and hyphae (BYE2). The data for all detected transcripts were plotted as Log_2_FC (expression of genes in BYE1/BYE2) versus the false discovery rate (FDR) of the adjusted *p*-value.

**Additional Fig. S3** Validation of the transcription analysis with the RNAseq by qRT-PCR for selected genes. *FAS1* and *ACC* encoding fatty-acid synthase complex protein and acetyl-CoA carboxylase for lipid biosynthesis (Fig. 4). *RAS2* and *TPK3* encoding the RAS GTPase and the catalytic subunit of PKA for dimorphic signaling through the cAMP-PKA pathway and *SSK1* encoding SSK1 for the two-component system mediating response to oxidative stress through the MAP Kinase pathway (Fig. 6). *TUP1* and *NRG1* encoding the transcriptional repressor TUP1 involving filamentous growth and the DNA-binding regulator binding to TUP1. *RIM 9* and *RIM 101* encoding transcription factors for response to pH variations. *GAL10* encoding the UDP-glucose 4-epimerase 2.


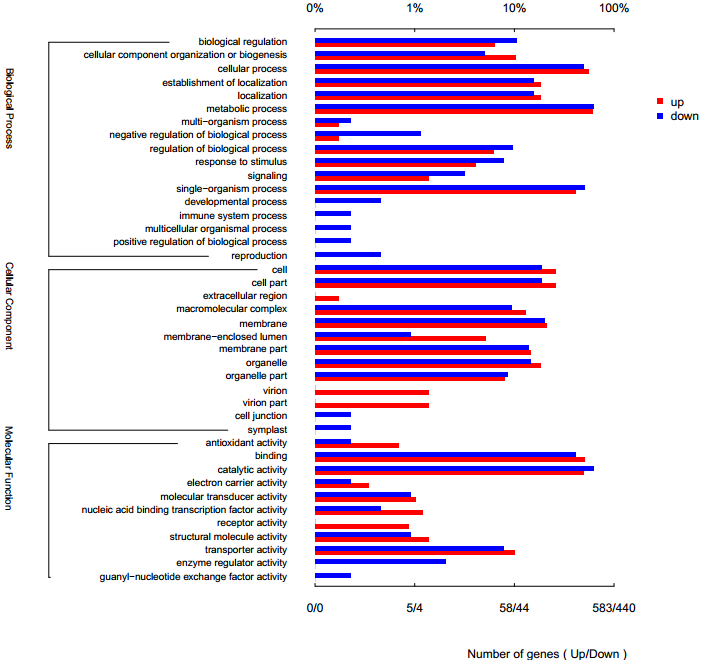


**Additional Fig. S4** GO classification for *T. cutaneum* B3 transcripts. Specific GO terms assigned to 36, 944 transcripts were grouped into three main categories: biological process, cellular component and molecular function.

**Additional Fig. S5** Enrichment with top 50 KEGG pathways for DEGs and genes annotated for *T. cutaneum* B3 transcripts.

**Additional Table S1** Primers used in this study

| Gene | Primer | Sequence (5’-3’) |
| --- | --- | --- |
| *FAS1* | RQ311_G13_2F | CGCAACAACGCCATCAAG |
|  | RQ311_G13_2R | GACTCGCCACAGGTGAAG |
| *ACC* | RQ311_G14_1F | CAACGCCATCTCGCTCTTC |
|  | RQ311_G14_1R | GCAGCCTTCTCCATCTTCTC |
| *RAS2* | RQ311_G3_1F | AAGACCATCGCCGCCTAC |
|  | RQ311_G3_1R | CCTCGTCCTTGCCGTAAAC |
| *TPK3* | RQ311_G5_1F | GGCGTCGTCAATCGTCTC |
|  | RQ311_G5_1R | CGGTGTGCTGTTCTGTCTC |
| *SSK1* | RQ311_G4_1F | GATGTCGCCTGCTTGAAGAG |
|  | RQ311_G4_1R | TGATTGGTCCTTGTGCTTGTC |
| *TUP1* | RQ311_G6_1F | ACACAAGCACCCTTCTCAATC |
|  | RQ311_G6_1R | CAACGCATCCGACACCTTC |
| *NRG1* | RQ311_G7_1F | GCCTCAAGTCGTCCAACAG |
|  | RQ311_G7_1R | GCATGTAACCGCCGTAAGG |
| *RIM9* | RQ311_G9_1F | CAGCCGCTCGTCCAGTAG |
|  | RQ311_G9_1R | CCTCAAGAACCGCCTCCAG |
| *RIM101* | RQ311_G8_1F | GCACGCATCTTCATACATTCTC |
|  | RQ311_G8_1R | GCCGATGGTCACTGCTTAG |
| *GAL10* | RQ311_G12_1F | CATCCCATCACAGTCACCATC |
|  | RQ311_G12_1R | CGTGCGAGCCAATGTATCC |
| *GAPDH* | RQ311_G15_1F | GACCGATACGACCGAAACC |
|  | RQ311_G15_1R | CTCTCGCCTCTTCCAACTTC |
